# LEMD2‐associated progeroid syndrome: Expanding the phenotype of the nuclear envelopathy caused by a defect in *LEMD2* gene

**DOI:** 10.1111/acel.14189

**Published:** 2024-05-16

**Authors:** Alyssia Matter, Christina Kaufman, Nadia Zürcher, Daniela Lenggenhager, Patrice Grehten, Deborah Bartholdi, Laura Horka, Johannes Häberle, Georgios Makris

**Affiliations:** ^1^ Division of Metabolism and Children's Research Center University Children's Hospital Zurich Zurich Switzerland; ^2^ Department of Pathology and Molecular Pathology University Hospital Zurich Zurich Switzerland; ^3^ Department of Diagnostic Imaging University Children's Hospital Zurich Zurich Switzerland; ^4^ Department of Human Genetics, Inselspital Bern University Hospital Bern Switzerland; ^5^ Department of Endocrinology, Diabetology and Clinical Nutrition University Hospital Zurich Zurich Switzerland

**Keywords:** insulin resistance, LEMD2, nuclear envelopathy, nuclear phenotype, progeroid syndrome, rare disease

## Abstract

Nuclear envelopathies are rare genetic diseases that compromise the integrity of the nuclear envelope. Patients with a defect in LEM domain nuclear envelope protein 2 (*LEMD2*) leading to LEMD2‐associated progeroid syndrome are exceedingly scarce in number, yet they exhibit shared clinical features including skeletal abnormalities and a prematurely‐aged appearance. Our study broadens the understanding of LEMD2‐associated progeroid syndrome by detailing its phenotypic and molecular characteristics in the first female and fourth reported case, highlighting a distinct impact on metabolic functions. The patient's history revealed growth delay, facial and skeletal abnormalities, and recurrent abdominal pain crises caused by hepatomegaly. Comparisons with the previously documented cases emphasized similarities in skeletal and facial features while showcasing unique variations, notably in cardiac and hepatic manifestations. In vitro experiments conducted on patient‐derived peripheral blood and urinary epithelial cells and LEMD2‐downregulated HepG2 cells confirmed abnormalities in the structure of the nuclear envelope in all three tissue‐types. Overall, our work offers a comprehensive profile of a patient with LEMD2‐related syndrome, emphasizing the hepatic involvement in the disease and broadening our understanding of clinical and molecular implications. This study not only contributes specific insights into LEMD2‐related conditions but also underscores potential therapeutic paths for disorders affecting nuclear envelope dynamics.

## Graphical abstract

This study delves into LEM domain nuclear envelope protein 2 (LEMD2)‐associated progeroid syndrome, elucidating its phenotypic and molecular aspects in the first female and fourth reported case. In vitro experiments using patient‐derived cells and LEMD2‐downregulated HepG2 cells revealed pronounced nuclear envelope abnormalities. Our findings contribute to a deeper understanding of the clinical and molecular implications of LEMD2‐associated progeroid syndrome, highlighting a distinct impact on metabolic functions.

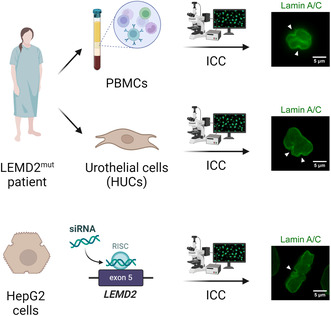

**Abbreviations**
AKT: protein kinase B
ALT: alanine transaminase
AST: aspartate transaminase
BAF: barrier‐to‐autointegration factor protein
EDMD: Emery–Dreifuss muscular dystrophy
ERK: extracellular signal‐regulated kinase
ESCRT‐III: endosomal sorting complex required for transport‐III
HGPS: Hutchinson–Gilford progeria syndrome
HUCs: human urothelial cells
INM: inner nuclear membrane
LEMD2: LEM domain nuclear envelope protein 2
MAPK: mitogen‐activated protein kinase
NE: nuclear envelope
PBMCs: human peripheral blood mononuclear cells
siCtrl: negative control siRNA
siLEMD2: LEMD2‐targeting siRNA
siRNA: small interfering RNA

## 1 INTRODUCTION, RESULTS, AND DISCUSSION

Nuclear envelopathies encompass a set of rare genetic conditions primarily affecting the structure and function of the nuclear envelope (NE) and the underlying lamina, resulting in various physiological manifestations (Somech et al., 2005). One prominent example is the envelopathy arising from mutations in *LMNA* gene, encoding lamin A and C via alternative splicing and resulting in diseases such as Hutchinson–Gilford progeria syndrome (OMIM #176670) and the autosomal forms of Emery–Dreifuss muscular dystrophy (OMIM #181350 and #616516) (Bonne & Quijano‐Roy, 2013; Ullrich & Gordon, 2015). These diseases present with diverse symptoms, including accelerated aging, muscle weakness, developmental delay, and skeletal and neurological abnormalities (Emery, 2000; Ullrich & Gordon, 2015).

LEM domain nuclear envelope protein 2 (LEMD2), a protein of the inner nuclear membrane (INM) and associated with the nuclear lamina, plays a crucial role in structural organization of the nucleus, NE integrity and its post‐mitotic restoration, similar to other INM proteins of the LEM‐domain family (Brachner et al., 2005; Ulbert et al., 2006). Studies suggest that LEMD2 also influences essential cellular signaling pathways and complexes including attenuation of the mitogen‐activated protein kinase (MAPK) and extracellular signal‐regulated kinase (ERK) cascades (Tapia et al., 2015), the protein kinase B (AKT) (Tapia et al., 2015), and the recruitment of endosomal sorting complex required for transport (ESCRTIII) essential for NE sealing and remodeling during cell division (Gu et al., 2017; von Appen et al., 2020). However, precise understanding of LEMD2 interplay and function in health and disease in humans remains rather limited.

**FIGURE 1:**
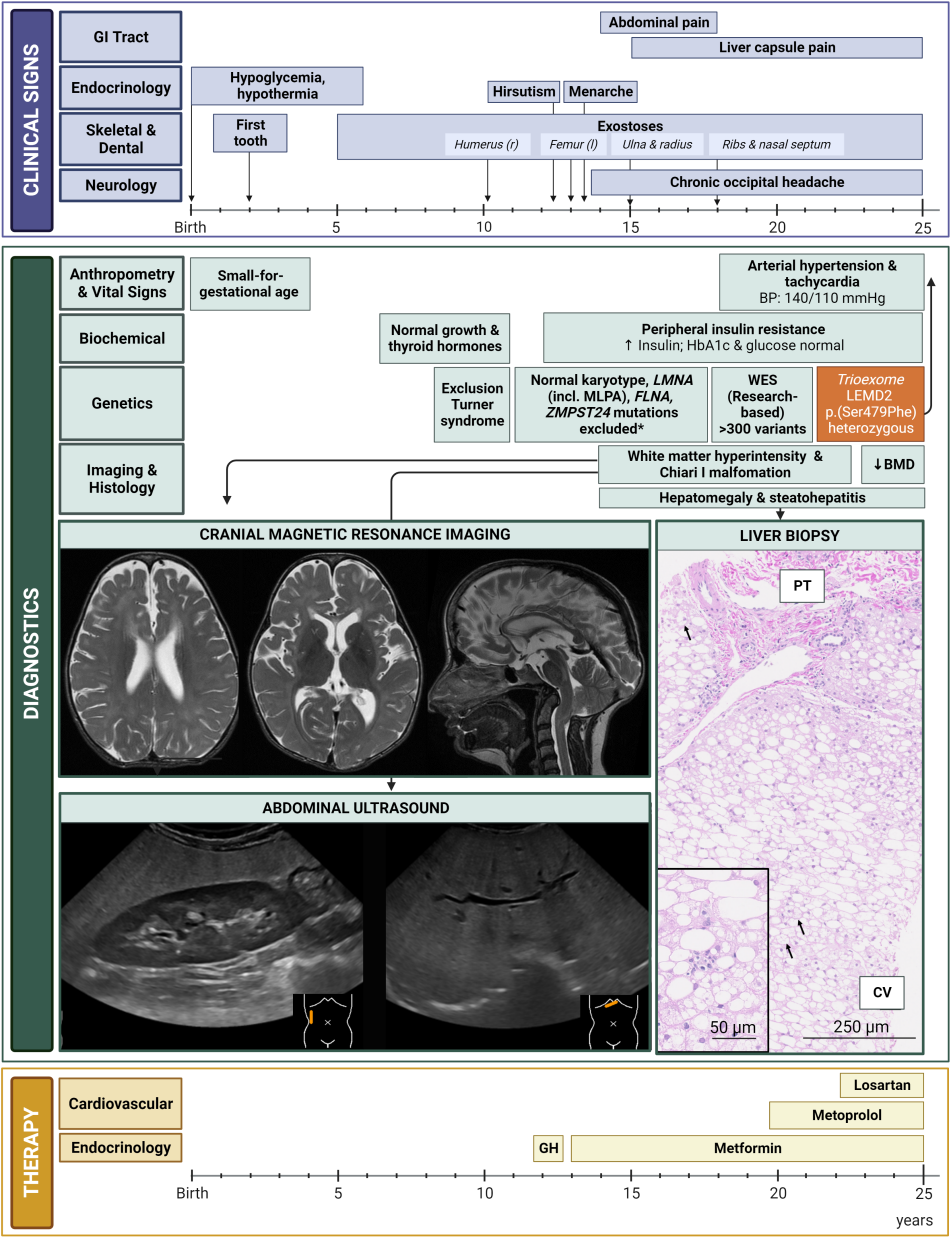
Time line (birth to age 25) depicting clinical signs, diagnostics and therapy in female patient with LEM domain nuclear envelope protein 2‐associated progeroid syndrome. Clinical signs are organized by affected organ system. Diagnostics include anthropometric data and vital signs, biochemical and genetic analyses, representative magnetic resonance imaging (MRI) of the brain, abdominal ultrasound and liver biopsy. Representative brain MRI at the age of 18 years. Axial T2 (left & middle panels): bilateral diffuse abnormal white matter hyperintensity with subcortical extension and predominance around the frontal horns. Sagittal T2 (right panel): Chiari I Malformation with platybasia and basilar invagination. Caudal displacement of the cerebellar tonsils and medulla oblongata through the foramen magnum with anterior impression of the medulla. Liver ultrasound with lateral (left) and subxyphoid (right) views. Hyperechogenicity of the liver parenchyma in comparison with the right kidney and hepatomegaly with extension of the right liver lobe inferior to the lower pole of the kidney. Liver biopsy (taken at the age of 14) with the following histological findings: massive macrovesicular (ca. 70%) and microvesicular (ca. 20%) steatosis, ballooned hepatocytes (arrows) and some necroinflammation (insert), consistent with steatohepatitis. Therapy section includes medication for cardiovascular and endocrinological manifestations. Pain medication is not shown. *Langer–Giedion syndrome and proximal 11p deletion syndrome were excluded. Created with BioRender.com. BMD, bone mineral density; BP, blood pressure; CV, central vein; GH, growth hormone; GI tract, gastrointestinal tract; MLPA, multiplex ligation‐dependent probe amplification; PT, portal tract; WES, whole‐exome sequencing.

Here, we present the first female and fourth overall patient with LEMD2‐associated nuclear envelopathy (Marbach‐Rustad progeroid syndrome, OMIM #619322) and aim to expand our current knowledge of this disease by examining her phenotypic characteristics as well as investigating novel clinical and molecular manifestations. The patient is the second offspring of healthy, non‐consanguineous Caucasian parents with a healthy older brother. Pregnancy was uneventful, and the patient was born in the 37th week of gestation with a low birth weight (2240 g, <P10), length (45 cm, <P10), and reduced head circumference (30.5 cm, <P3) (Figure 1; Figure S1). Only three individuals have so far been reported with a LEMD2‐associated nuclear envelopathy and all share interchangeable, progeria‐like physical and neurological manifestations (Lu et al., 2023; Marbach et al., 2019). In all three so far reported patients, the same heterozygous de novo c.1436C>T (p.Ser479Phe) pathogenic *LEMD2* variant was identified, which was also found in our patient in 2022 applying trio‐exome analysis. Notably, the published patients exhibited short stature, distinct facial features (triangular face, prominent eyes, mandibular hypoplasia) and dental abnormalities (delayed dentition, supernumerary teeth) (Table S1). In contrast to the published cases, our patient exhibited multiple and multilocal exostoses requiring repeated surgical removal since the age of 10 years, and no signs of intention tremor (Figure 1). Furthermore, beginning at the age of 14 years, our patient uniquely experienced recurring episodes of severe abdominal pain associated with hepatomegaly likely resulting from insulin resistance and liver steatosis, as seen by alanine and aspartate transaminases, and regularly required intensified pain management with opioids, cannabis as well as metformin for treatment of peripheral insulin resistance (Figure 1; Figure S2A,B; Table S2). Of note, metformin demonstrated dose‐dependent effects in terms of insulin regulation, since administration of 2000 mg/day or above contributed to insulin depression close to reference range (Figure S2B). The hepatic phenotype seems to be a relevant clinical sign also in other laminopathies, such as those triggered by *LMNA* mutations. It is known that *lmna* knockout in mice promotes spontaneous development of hepatic steatosis, predominantly affecting male animals and showcasing heightened susceptibility to steatohepatitis and fibrosis induced by high‐fat diet (Kwan et al., 2017). Conversely, patients with metabolic syndrome have been reported to exhibit undetected laminopathies characterized by hepatic steatosis, high blood pressure, and triglyceride accumulation (Dutour et al., 2011). As an adult, our patient interestingly developed arterial hypertension and tachycardia, which were treated with metoprolol and later losartan (Supplementary Material, “Detailed patient presentation, clinical course & treatment”) as well as a sustained increase in plasma triglycerides (Figure S2C; Table S2). These symptoms are frequently observed in other progeroid syndromes (Garg et al., 2009; Hussain et al., 2018; Merideth et al., 2008) and indicate a potential role for LEMD2 in hepatic homeostasis regulation, a function already proposed for lamin A/C (Kwan et al., 2017). During the same developmental stage, we detected in our patient elevated activity of serum biotinidase (Figure S2C; Table S2). This enzyme is responsible for the recycling and bioavailability of biotin in the human body (Zempleni et al., 2008) and, if elevated, acts as an indicator for upregulation of gluconeogenesis and fatty acid synthesis with yet not entirely understood pathophysiology (Forny et al., 2021). This atypical presentation was not observed so far in LEMD2‐associated progeroid syndrome or other envelopathies and its potential significance as a LEMD2‐associated metabolic clue remains unclear.

**FIGURE 2:**
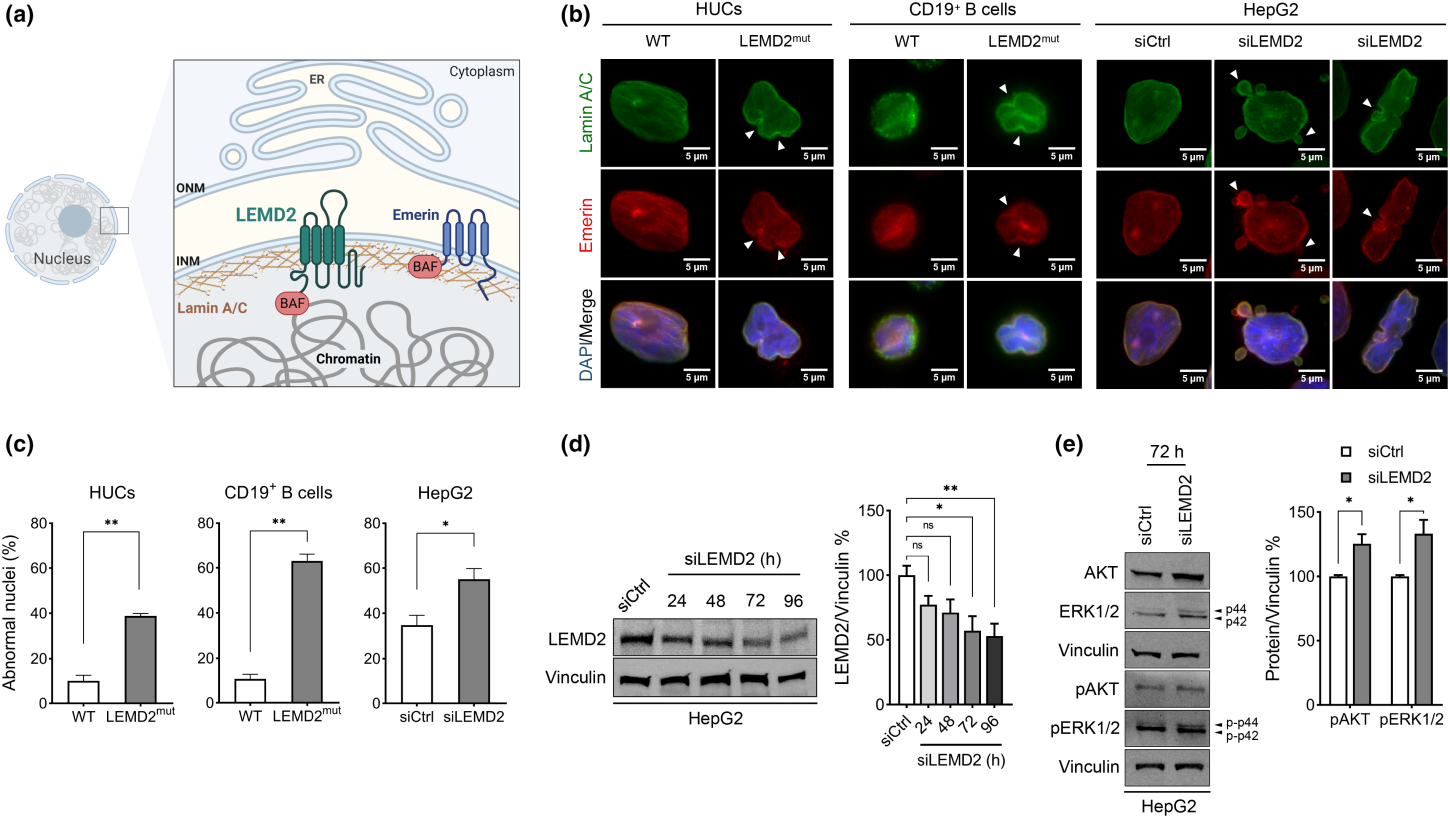
Molecular characterization of LEM domain nuclear envelope protein 2 (LEMD2)‐associated progeroid syndrome. (a) Simplified schematic of nuclear envelope (NE) structure and proteins, including LEMD2, emerin, and lamin A/C. The inner and outer nuclear membranes (INM, ONM) as well as the endoplasmic reticulum (ER) are indicated. Interaction of NE with chromatin is facilitated through barrier‐to‐autointegration factor protein. Created with BioRender.com. (b) Representative immunocytochemistry images of nuclei from LEMD2 patient‐derived human urothelial cells (HUCs), CD19^+^ B cells and HepG2 cell subjected to siRNA‐mediated LEMD2 knockdown for 72 h. HUC and CD19^+^ B‐cell nuclei from a healthy female donor or siCtrl‐treated HepG2 cells were included as controls. White arrows point to nuclear abnormalities (invaginations, blebbing). Scale bars = 5 μΜ. (c) Abnormal nuclei quantification graphs. Quantification of nuclei from HUCs (WT, *n* = 130; LEMD2^mut^, *n* = 115), CD19^+^ B cells (WT, *n* = 242; LEMD2^mut^, *n* = 217), and HepG2 cells (siCtrl, *n* = 219; siLEMD2, *n* = 236) was performed. Mean ± SD of three independent experiments (*t* test, *p ≤ 0.05, **p ≤ 0.01). (d) Representative western blot and band intensity quantification graph for monitoring the siRNA‐mediated LEMD2 knockdown up to 96 h post‐transfection. SiCtrl‐treated HepG2 cells served as control. Mean ± SD of three independent experiments (one‐way ANOVA with Dunnett's multiple comparisons test, ns = non‐significant, *p ≤ 0.05, **p ≤ 0.01). (e) Representative western blot and band intensity quantification graph for monitoring expression of AKT, ERK1/2, and pAKT and pERK1/2 in HepG2 cells at 72 h post‐transfection with either siCtrl or siLEMD2. Quantification performed for the phosphorylated proteins. Mean ± SD of three independent experiments (*t* test, *p ≤ 0.05).

LEMD2 as part of the INM (Figure 2a) functionally and physically interacts with lamin A/C and shares a 40 amino acid nucleoplasmic motif termed LEM‐domain with other unrelated INM proteins including emerin and MAN1 (Brachner et al., 2005; Wagner & Krohne, 2007). Moreover, LEMD2 associates with chromatin through the barrier‐to‐autointegration factor protein (Chen et al., 2023). This interaction contributes to the maintenance of heterochromatin, resembling the repressive chromatin mechanisms facilitated by emerin, lamin A, lamin B, and LAP2 complex (Chi et al., 2009). To examine the manifestation of LEMD2‐associated nuclear envelopathy on molecular level, we probed the expression pattern of lamin A/C and emerin in patient‐derived urothelial cells (HUCs) and CD19^+^ B lymphocytes. Using immunocytochemistry, we revealed that a significant proportion of both HUCs and CD19^+^ B cells exhibited altered nuclear morphology, when compared to cell nuclei from a healthy donor (Figure 2b,c; Figure S3). The immunofluorescent staining for both lamin A/C and emerin pinpointed irregular NE shaping, characteristic membrane invaginations and intrusions and loss of circular morphology with albeit proper nuclear localization. This discovery broadens the molecular and phenotypic understanding by utilizing cell models more pertinent than the skin fibroblast cells from the initial reported patients with LEMD2‐associated progeroid syndrome (Marbach et al., 2019). It also aligns with earlier in vitro investigations indicating that HeLa and U2OS cells lacking *lem2*, an alternative alias for *LEMD2* gene, exhibited nuclei with abnormal shapes (Ulbert et al., 2006). Alternatively, one study demonstrated reduced incidence of unfolded nuclei in hippocampal neurons upon LEMD2 knockdown, however only concomitant to SATB2 overexpression, a DNA‐binding protein implicated with chromatin remodeling and cognitive function regulation (Feurle et al., 2021). To further interrogate these findings and since our patient exhibited hepatic manifestations, we utilized HepG2 cells as surrogate hepatic model and performed siRNA‐mediated LEMD2 knockdown. LEMD2 depression and nuclei morphology were evaluated at 72 h post‐transfection. At this time point, a protein knockdown in the range of 50% was achieved (Figure 2d), mimicking the autosomal dominant form of LEMD2‐associated nuclear envelopathy with one functional and one diseased allele. The nuclei of siLEMD2‐treated HepG2 cells demonstrated irregular shapes and NE invaginations when compared to siCtrl‐treated cells, as indicated by lamin A/C and emerin staining (Figure 2b,c; Figure S3). In some instances, we observed additional nuclear blebbing otherwise commonly associated with apoptotic cells (Coleman et al., 2001). Indeed, previous results in HeLa cells indicated reduced cell proliferation or even cellular death upon siRNA‐mediated LEMD2 depletion (Ulbert et al., 2006).

Apart from its mechanistic role in supporting NE integrity, LEMD2 has been implicated with embryogenesis and cardiac development (Tapia et al., 2015). Indeed, mice lacking *lem2* expression were embryonic lethal and cardiomyocytes from *lem2*‐depleted animal hearts exhibited nuclear abnormalities and engaged programmed cell death (Ross et al., 2023). Alternatively, engineering mice with the human c.38T>G (p.Leu13Arg) *Lemd2* variant resulted in severe dilated cardiomyopathy, cardiac fibrosis, and premature death, along with the identification of nuclear deformations in isolated cardiomyocytes from these animals (Caravia et al., 2022). These observations contribute to unraveling the phenotypic complexity in our patient, who, already in early adulthood, presented with arterial hypertension, a dilated, eccentric hypertrophic left ventricle, and a fibrotic aortic valve with mild insufficiency and stenosis (Supplementary Material, “Detailed patient presentation, clinical course & treatment”), symptoms that were not previously documented (Marbach et al., 2019).

Furthermore, LEMD2 is involved in regulation of central signaling cascades such as MAPK/ERK and AKT pathways, since LEMD2 loss of function enhanced MAP kinase and AKT phosphorylation and activation in animal models (Ross et al., 2023; Tapia et al., 2015). In this context, we performed experiments with siLEMD2‐treated HepG2 cells and confirmed increased and sustained phosphorylation of pAKT and pERK1/2 at 72 h post‐transfection or longer, in contrast to control‐treated cells (Figure 2e; Figure S4). Yet, future work on better understanding and deciphering the role of LEMD2 on MAPK/ERK and AKT signaling pathways is required. As such, employing a hepatic cell model such as the HepaRG cells, which exhibit metabolic characteristics similar to primary human hepatocytes, the established hepatic gold standard, would be advantageous (Makris et al., 2024).

While acknowledging the constraints inherent in a study based on a single patient and limited literature, our research illustrates novel aspects of LEMD2‐associated progeroid syndrome, including the distinct clinical features concerning the cardiac manifestations and steatohepatitis with associated hepatomegaly resulting in liver capsule pain. By conducting thorough clinical assessments and cell culture studies, we broadened our understanding of LEMD2‐related nuclear envelopathy. Our investigations confirmed the existence of previously identified nuclear membrane irregularities and established the involvement of MAPK and AKT pathways in patient‐derived immune cells, urine‐derived cells, and a hepatocyte surrogate model. These efforts are crucial in advancing our comprehension of LEMD2 as a genetic element influencing human health, thereby enhancing awareness within the medical community.

## AUTHOR CONTRIBUTIONS

Christina Kaufman, Johannes Häberle, and Georgios Makris designed the study. Alyssia Matter, Christina Kaufman, Nadia Zürcher, and Georgios Makris performed experiments and researched and analyzed data. Daniela Lenggenhager, Patrice Grehten, Deborah Bartholdi, and Laura Horka contributed specific clinical data. Alyssia Matter, Christina Kaufman, Johannes Häberle, and Georgios Makris wrote the manuscript. All authors read and approved the final manuscript.

## FUNDING INFORMATION

This research received no specific grant from any funding agency in the public, commercial, or not‐for‐profit sectors.

## CONFLICT OF INTEREST STATEMENT

The authors declare no conflict of interest.

## PERMISSION STATEMENT

The study protocol in this single patient did not require specific approval by the Swissethics committee according to Swiss regulations. The patient cells were obtained with written informed consent of the respective individual.

## Supporting information


Figure S1.
Figure S2.
Figure S3.
Figure S4.
Table S1.
Table S2.

## Data Availability

All data generated or analyzed during this study are included in this published article (and its supporting information file).

## References

[acel14189-bib-0001] Bonne, G. , & Quijano‐Roy, S. (2013). Emery‐Dreifuss muscular dystrophy, laminopathies, and other nuclear envelopathies. Pediatric Neurology, 113, 1367–1376. 10.1016/B978-0-444-59565-2.00007-1 23622360

[acel14189-bib-0002] Brachner, A. , Reipert, S. , Foisner, R. , & Gotzmann, J. (2005). LEM2 is a novel MAN1‐related inner nuclear membrane protein associated with A‐type lamins. Journal of Cell Science, 118(24), 5797–5810. 10.1242/jcs.02701 16339967

[acel14189-bib-0003] Caravia, X. M. , Ramirez‐Martinez, A. , Gan, P. H. , Wang, F. , McAnally, J. R. , Xu, L. , Bassel‐Duby, R. , Liu, N. , & Olson, E. N. (2022). Loss of function of the nuclear envelope protein LEMD2 causes DNA damage‐dependent cardiomyopathy. Journal of Clinical Investigation, 132(22). 10.1172/JCI158897 PMC9663152 36377660

[acel14189-bib-0004] Chen, R. P. , Buchmann, S. , Kroth, A. , Arias‐Loza, A. P. , Kohlhaas, M. , Wagner, N. , Grüner, G. , Nickel, A. , Cirnu, A. , Williams, T. , Maack, C. , Ergün, S. , Frantz, S. , & Gerull, B. (2023). Mechanistic insights of the LEMD2 p.L13R mutation and its role in cardiomyopathy. Circulation Research, 132(2), e43–e58. 10.1161/Circresaha.122.321929 36656972

[acel14189-bib-0005] Chi, Y. H. , Chen, Z. J. , & Jeang, K. T. (2009). The nuclear envelopathies and human diseases. Journal of Biomedical Science, 16, 96. 10.1186/1423-0127-16-96 19849840 PMC2770040

[acel14189-bib-0006] Coleman, M. L. , Sahai, E. A. , Yeo, M. , Bosch, M. , Dewar, A. , & Olson, M. F. (2001). Membrane blebbing during apoptosis results from caspase‐mediated activation of ROCK I. Nature Cell Biology, 3(4), 339–345. 10.1038/35070009 11283606

[acel14189-bib-0007] Dutour, A. , Roll, P. , Gaborit, B. , Courrier, S. , Alessi, M. C. , Tregouet, D. A. , Angelis, F. , Robaglia‐Schlupp, A. , Lesavre, N. , Cau, P. , Lévy, N. , Badens, C. , & Morange, P. E. (2011). High prevalence of laminopathies among patients with metabolic syndrome. Human Molecular Genetics, 20(19), 3779–3786. 10.1093/hmg/ddr294 21724554

[acel14189-bib-0008] Emery, A. E. H. (2000). Emery‐Dreifuss muscular dystrophy—A 40 year retrospective. Neuromuscular Disorders, 10(4–5), 228–232. 10.1016/S0960-8966(00)00105-X 10838246

[acel14189-bib-0009] Feurle, P. , Abentung, A. , Cera, I. , Wahl, N. , Ablinger, C. , Bucher, M. , Stefan, E. , Sprenger, S. , Teis, D. , Fischer, A. , Laighneach, A. , Whitton, L. , Morris, D. W. , Apostolova, G. , & Dechant, G. (2021). SATB2‐LEMD2 interaction links nuclear shape plasticity to regulation of cognition‐related genes. EMBO Journal, 40(3), e103701. 10.15252/embj.2019103701 33319920 PMC7849313

[acel14189-bib-0010] Forny, P. , Burda, P. , Bode, P. , & Rohrbach, M. (2021). Is serum biotinidase enzyme activity a potential marker of perturbed glucose and lipid metabolism? JIMD Reports, 57(1), 58–66. 10.1002/jmd2.12168 33473341 PMC7802622

[acel14189-bib-0011] Garg, A. , Subramanyam, L. , Agarwal, A. K. , Simha, V. , Levine, B. , D'Apice, M. R. , Novelli, G. , & Crow, Y. (2009). Atypical progeroid syndrome due to heterozygous missense mutations. The Journal of Clinical Endocrinology and Metabolism, 94(12), 4971–4983. 10.1210/jc.2009-0472 19875478 PMC2795646

[acel14189-bib-0012] Gu, M. Y. , LaJoie, D. , Chen, O. S. , von Appen, A. , Ladinsky, M. S. , Redd, M. J. , Nikolova, L. , Bjorkman, P. J. , Sundquist, W. I. , Ullman, K. S. , & Frost, A. (2017). LEM2 recruits CHMP7 for ESCRT‐mediated nuclear envelope closure in fission yeast and human cells. Proceedings of the National Academy of Sciences of the United States of America, 114(11), E2166–E2175. 10.1073/pnas.1613916114 28242692 PMC5358359

[acel14189-bib-0013] Hussain, I. , Patni, N. , Ueda, M. , Sorkina, E. , Valerio, C. M. , Cochran, E. , Brown, R. J. , Peeden, J. , Tikhonovich, Y. , Tiulpakov, A. , Stender, S. R. S. , Klouda, E. , Tayeh, M. K. , Innis, J. W. , Meyer, A. , Lal, P. , Godoy‐Matos, A. F. , Teles, M. G. , Adams‐Huet, B. , … Garg, A. (2018). A novel generalized lipodystrophy‐associated progeroid syndrome due to recurrent heterozygous p.T10I mutation. The Journal of Clinical Endocrinology and Metabolism, 103(3), 1005–1014. 10.1210/jc.2017-02078 29267953 PMC6283411

[acel14189-bib-0014] Kwan, R. , Brady, G. F. , Brzozowski, M. , Weerasinghe, S. V. , Martin, H. , Park, M. J. , Brunt, M. J. , Menon, R. K. , Tong, X. , Yin, L. , Stewart, C. L. , & Omary, M. B. (2017). Hepatocyte‐specific deletion of mouse lamin A/C leads to male‐selective steatohepatitis. Cellular and Molecular Gastroenterology and Hepatology, 4(3), 365–383. 10.1016/j.jcmgh.2017.06.005 28913408 PMC5582719

[acel14189-bib-0015] Lu, Z. K. , Zhang, W. , Mao, X. J. , Li, D. , Chen, X. D. , Liu, L. , & Lin, Y. T. (2023). The third case of Marbach‐Rustad progeroid syndrome caused by a de novo LEMD2 variant. Clinical Genetics, 105, 209–213. 10.1111/cge.14441 37867468

[acel14189-bib-0016] Makris, G. , Veit, L. , Rüfenacht, V. , Klassa, S. , Zürcher, N. , Matsumoto, S. , Poms, M. , & Häberle, J. (2024). Expression and function of the urea cycle in widely‐used hepatic cellular models. Journal of Inherited Metabolic Disease. 10.1002/jimd.12701 38192032

[acel14189-bib-0017] Marbach, F. , Rustad, C. F. , Riess, A. , Đukić, D. , Hsieh, T. C. , Jobani, I. , Prescott, T. , Bevot, A. , Erger, F. , Houge, G. , Redfors, M. , Altmueller, J. , Stokowy, T. , Gilissen, C. , Kubisch, C. , Scarano, E. , Mazzanti, L. , Fiskerstrand, T. , Krawitz, P. M. , … Netzer, C. (2019). The discovery of a LEMD2‐associated nuclear envelopathy with early progeroid appearance suggests advanced applications for AI‐driven facial phenotyping. American Journal of Human Genetics, 104(4), 749–757. 10.1016/j.ajhg.2019.02.021 30905398 PMC6451726

[acel14189-bib-0018] Merideth, M. A. , Gordon, L. B. , Clauss, S. , Sachdev, V. , Smith, A. C. M. , Perry, M. B. , Brewer, C. C. , Zalewski, C. , Kim, H. J. , Solomon, B. , Brooks, B. P. , Gerber, L. H. , Turner, M. L. , Domingo, D. L. , Hart, T. C. , Graf, J. , Reynolds, J. C. , Gropman, A. , Yanovski, J. A. , … Introne, W. J. (2008). Phenotype and course of Hutchinson‐Gilford progeria syndrome. New England Journal of Medicine, 358(6), 592–604. 10.1056/NEJMoa0706898 18256394 PMC2940940

[acel14189-bib-0019] Ross, J. A. , Arcos‐Villacis, N. , Battey, E. , Boogerd, C. , Orellana, C. A. , Marhuenda, E. , Swiatlowska, P. , Hodzic, D. , Prin, F. , Mohun, T. , Catibog, N. , Tapia, O. , Gerace, L. , Iskratsch, T. , Shah, A. M. , & Stroud, M. J. (2023). Lem2 is essential for cardiac development by maintaining nuclear integrity. Cardiovascular Research, 119(11), 2074–2088. 10.1093/cvr/cvad061 37067297 PMC10478753

[acel14189-bib-0020] Somech, R. , Shaklai, S. , Amariglio, N. , Rechavi, G. , & Simon, A. J. (2005). Nuclear envelopathies–raising the nuclear veil. Pediatric Research, 57(5 Pt 2), 8R–15R. 10.1203/01.PDR.0000159566.54287.6C 15817509

[acel14189-bib-0021] Tapia, O. , Fong, L. G. , Huber, M. D. , Young, S. G. , & Gerace, L. (2015). Nuclear envelope protein Lem2 is required for mouse development and regulates MAP and AKT kinases. PLoS One, 10(3), e0116196. 10.1371/journal.pone.0116196 25790465 PMC4366207

[acel14189-bib-0022] Ulbert, S. , Antonin, W. , Platani, M. , & Mattaj, I. W. (2006). The inner nuclear membrane protein Lem2 is critical for normal nuclear envelope morphology. FEBS Letters, 580(27), 6435–6441. 10.1016/j.febslet.2006.10.060 17097643

[acel14189-bib-0023] Ullrich, N. J. , & Gordon, L. B. (2015). Hutchinson‐Gilford progeria syndrome. Handbook of Clinical Neurology, 132, 249–264. 10.1016/B978-0-444-62702-5.00018-4 26564085

[acel14189-bib-0024] von Appen, A. , LaJoie, D. , Johnson, I. E. , Trnka, M. J. , Pick, S. M. , Burlingame, A. L. , Ullman, K. S. , & Frost, A. (2020). LEM2 phase separation promotes ESCRT‐mediated nuclear envelope reformation. Nature, 582(7810), 115–118. 10.1038/s41586-020-2232-x 32494070 PMC7321842

[acel14189-bib-0025] Wagner, N. , & Krohne, G. (2007). LEM‐domain proteins: New insights into lamin‐interacting proteins. International Review of Cytology—A Survey of Cell Biology, 261(261), 1–46. 10.1016/S0074-7696(07)61001-8 17560279

[acel14189-bib-0026] Zempleni, J. , Hassan, Y. I. , & Wijeratne, S. S. (2008). Biotin and biotinidase deficiency. Expert Review of Endocrinology and Metabolism, 3(6), 715–724. 10.1586/17446651.3.6.715 19727438 PMC2726758

