# Supplementary material for: LEMD2‐associated progeroid syndrome: Expanding the phenotype of the nuclear envelopathy caused by a defect in LEMD2 gene
Source: Aging Cell. 2024 May 16;23(8):e14189. doi: 10.1111/acel.14189 (PMC11320348; doi:10.1111/acel.14189)

**LEMD2-associated progeroid syndrome: expanding the phenotype of the nuclear envelopathy caused by a defect in *LEMD2* gene**

**Running title:** Fourth LEMD2-mutant patient depiction

Alyssia Matter^1,*^, Christina Kaufman^1,*^, Nadia Zürcher^1^, Daniela Lenggenhager^2^, Patrice Grehten^3^, Deborah Bartholdi^4^, Laura Horka^5^, Johannes Häberle^1^, Georgios Makris^1^

^1^Division of Metabolism and Children’s Research Center, University Children’s Hospital Zurich, Zurich, Switzerland

^2^Department of Pathology and Molecular Pathology, University Hospital Zurich, Zurich, Switzerland

^3^Department of Diagnostic Imaging, University Children’s Hospital Zurich, Zurich, Switzerland

^4^Department of Human Genetics, Inselspital, Bern University Hospital, Bern, Switzerland

^5^Department of Endocrinology, Diabetology and Clinical Nutrition, University Hospital Zurich, Zurich, Switzerland

alyssia.matter@uzh.ch

christina.kaufman@kispi.uzh.ch

nadia.zuercher@kispi.uzh.ch

daniela.lenggenhager@usz.ch

patrice.grehten@kispi.uzh.ch

deborah.bartholdi@insel.ch

laura.horka@usz.ch

johannes.haeberle@kispi.uzh.ch

^*^Alyssia Matter and Christina Kaufman contributed equally.

**Correspondence**

Georgios Makris, Division of Metabolism and Children’s Research Center, University Children’s Hospital Zurich, Steinwiesstrasse 75, CH-8032, Zurich, Switzerland Email: georgios.makris@kispi.uzh.ch

**Supplementary Material**

- **Detailed patient presentation, clinical course & treatment**
- **Experimental procedures**
- **Tables S1-S2**
- **Figure S1**
- **Figure S2**

**Detailed patient presentation, clinical course & treatment**

**Early Years**

The individual was born in the 37^th^ week of gestation after an uneventful pregnancy, weighing 2240 g (< P10), with a length of 45 cm (< P10) and head circumference of 30.5 cm (< P10). The newborn experienced hypoglycemia (level not documented) and hypothermia (level not documented). In infancy at the age of 10 months, the patient faced growth challenges and failure to thrive, with her weight falling below the 3^rd^ percentile.

**Skull & Dentures**

At birth, the individual exhibited wide skull sutures and microcephaly. Tooth development was significantly delayed, with the first tooth appearing at the age of 4 years. By the age of 6 years, only 5 deciduous teeth had erupted. X-rays of the jaw revealed that all teeth were present within the gums. At age 20 years, she had reconstruction and augmentation of the maxilla through total onlay grafting (16 tooth extractions from the upper jaw, 4 prosthetic dental implants for the lower jaw).

**Skeletal Features**

From the age of 5 years onward, the patient experienced cartilage exostoses in multiple bones (distal femur, nasal bone, several ribs, distal radius and ulna, and clavicles), partly necessitating surgical removal due to pain, motor, or sensory deficits. The individual exhibited skeletal dysplasia of unknown origin with short stature. During adolescence, she suffered from vitamin D deficiency, which was treated with vitamin D and calcium supplementation. At 23 years, osteodensitometry showed low bone density of the hip (z-score: -2.6) and femoral neck (z-score: -3.1). MR imaging of the spine at age 15 years showed hyperlordosis, lumbosacral transitional vertebra with lumbarization of S1 with spondylolisthesis and an anterior translation of 3 mm with consecutive foraminal stenosis on the left side. At the age of 16 years, CT-guided facet joint infiltration at levels L4/5 and L5/S1 was performed on both sides, with limited success. *In situ* spondylodesis L4-S1 was successfully performed 3 months later resulting in an immediate and persistent pain reduction.

**Endocrinology**

At the age of 13 years, the patient started growth hormone therapy, resulting in a small temporary growth increase. However, the overall growth rate continued to decline. The therapy was discontinued after 6 months due to onset of peripheral insulin resistance, which was subsequently treated with metformin. Ultimately, growth hormone therapy and insulin resistance were associated with elevated testosterone levels and hirsutism. Hyperinsulinism persisted ever since age 14, averaging at 397.9 pmol/L (reference 36-150).

**Gastrointestinal**

At age 14, the individual was diagnosed with non-erosive antrum gastritis through gastroscopy and was prescribed proton pump inhibitors (esomeprazole 40 mg/d). At age 15, the patient was hospitalized for a month due to persistent abdominal pain, emesis, and loss of appetite that had lasted for 6 months. An abdominal ultrasound revealed massive hepatomegaly with small zones of reduced perfusion as the likely cause of her liver capsule pain. Liver biopsies showed massive macrovesicular (ca. 70%) and microvesicular (ca. 20%) steatosis, ballooned hepatocytes and some necroinflammation, consistent with steatohepatitis. The patient's hepatic symptoms continued throughout the years with occasional additional pruritus. Symptom management included buprenorphine patches and later medical cannabis. Liver parameters GGT (gamma-glutamyltransferase), ALP (alkaline phosphatase), AST (aspartate transaminase), and ALT (alanine transaminase) were persistently increased in plasma, along with elevated plasma triglycerides and serum biotinidase (Figure 1, Table S2).

**Reproductive System**

Upon metformin treatment, insulin resistance was regulated, while hyperandrogenism was reduced, resulting in the individual's first menstruation (menarche) at age 13 years 2 months. At age 14, polycystic ovary syndrome (PCOS) was suspected, which was later confirmed. She underwent a right laparoscopic ovarian cystectomy at age 14 years and experienced an ovarian cyst rupture 2 years later.

**Neurology**

The individual's motor and cognitive functions were normal, except for some memory difficulties. Due to bilateral occipital headaches, a neurological assessment was conducted when she was 14 years old. This included a cranial MRI, which revealed signs of dysplasia of the skull base, Chiari-I malformation, base impressions, and pronounced white matter hyperintensities. Additionally, the patient experienced vision impairment and occipital neuralgia at age 18, and one year later suffered from an episode of transient aphasia.

**Anthropometry**

At 23 years, her length was 131 cm (< P3), and she weighed 27 kg (< P3) resulting in a BMI of 15.2 kg/m^2^.

**Cardiovascular**

At age 18, the patient developed arterial hypertension and tachycardia (BP 140/110 mmHg, heart rate 130/min), which were managed with beta-blockers (metoprolol 25 mg/d). She was found to have a dilated, eccentric hypertrophic left ventricle with a low to normal ejection fraction (EF, 47%), and a highly fibrotic aortic valve with light insufficiency and stenosis. At age 20, exertional dyspnea NYHA II-III was diagnosed, and the patient was prescribed an AT1-receptor antagonist (losartan; before ACE inhibitors were not well tolerated).

**Social Life**

The individual lives an independent life. She successfully completed an apprenticeship as a photographer and performed well in school, attending four days per week (three full days and two half days).

**Diagnosis**

At age 25, the individual received the diagnosis of LEMD2-associated nuclear envelopathy (Marbach-Rustad progeroid syndrome, OMIM #619322) following whole exome sequencing that identified the *de novo* c.1436C>T; p.Ser479Phe pathogenic, heterozygous mutation in the *LEMD2* gene.

**Experimental procedures**

**Preparation of peripheral blood mononuclear cells (PBMCs)**

For PBMC preparation, blood (5 mL) was withdrawn either from the patient or a healthy individual. The blood sample was treated with EDTA, diluted 1:1 with phosphate-buffered saline (PBS) into a final volume of 10 mL and then overlaid on Histopaque®-1077 density gradient medium (10771, Sigma). Tubes were centrifuged at 400xg for 30 min at room temperature. Four (4) layers (plasma, PBMCs, gradient medium and red blood cells (RBCs)) were observed and the PBMC layer was then isolated, washed with PBS, and centrifuged sequentially, each time removing excess plasma, platelets, and gradient medium. The cells were then resuspended in 2 mL RPMI 1640 medium (11875093, ThermoFisher) and counted using XN-350 automated hematology analyzer (Sysmex).

**Isolation of CD19^+^ B-lymphocytes**

CD19 microBeads (130-050-301, Miltenyi Biotec) were employed to isolate B-lymphocytes from the PBMC fraction. Briefly, PBMC preparation was resuspended in MACS® isolation buffer containing 1X PBS, 2% fetal bovine serum (FBS) (26140079, ThermoFisher) and 2 mM EDTA. CD19 MicroBeads were added per 10^7^ cells, following 15 min incubation at 4°C. After two sequential washing and centrifugation steps, B-lymphocytes were isolated in positive selection mode using autoMACS® Pro Separator (Miltenyi Biotec) using MACS® isolation buffer (130-091-221, Miltenyi Biotec).

**Urothelial cell culture**

Patient-derived or age-matched healthy female urothelial cells were isolated using a previously described method (Erden Tayhan et al. 2017), with slight modification. Briefly, after collection, the urinary sample was immediately transferred to the cell culture laboratory and kept at 4°C. The sample was centrifuged at 500xg for 5 min, resuspended in PBS solution and then centrifuged once again. Supernatant was discarded and the cells were collected with initiation medium composed of 1:1 mixture of Keratinocyte serum-free medium (KSFM) (17005042, ThermoFisher) and embryonic fibroblast medium (EFM). EFM contained DMEM (11965092, ThermoFisher) and Ham’s F12 (11765054, ThermoFisher) in the ratio of 3:1, respectively. The initiation medium also contained 10% FBS, 0.4 µg/mL hydrocortisone (H2270, Sigma), 0.1 mM non-essential amino-acids (NEAAs) (11140035, ThermoFisher), 0.01% insulin (12585014, ThermoFisher) and 1% penicillin-streptomycin (15070063, ThermoFisher). Cells were incubated at 37°C in 5% CO_2_. Cell culture medium was replaced every 2-3 days and cells were passaged at 80% confluency.

**HepG2 cell culture**

HepG2 cells (HB-8065TM, ATCC) were maintained in RMPI 1640 supplemented with 10% FBS and 1% penicillin-streptomycin (15070063, ThermoFisher). Medium changes were performed every 2-3 days and cells were passaged at 80% confluency. Cell cultures were incubated at 37°C in a humidified atmosphere containing 5% CO_2_. For knockdown and immunostaining experiments, 1×10^5^ HepG2 cells were seeded into glass microscope cover slips and were left to attach for 24 h prior to experiment initiation.

**siRNA transfection**

The patient holds a heterozygous mutation for *LEMD2* (c.1436C>T; p.Ser479Phe). To mimic a reduced LEMD2 functionality, HepG2 cells were transfected with Silencer® Select siRNA (AM16708, ThermoFisher) against LEMD2 using Lipofectamine™ RNAiMAX Transfection Reagent (13778030, ThermoFisher). Wells transfected with Silencer™ Negative Control No. 1 siRNA (AM4611, ThermoFisher) were included. Time-course knockdown experiments indicated that ~50% protein reduction was achieved at 72-96 h post-transfection. All siRNAs were used at a final concentration of 1 × 10^-4^ mmol/L.

**Western Blotting analysis**

Cells were lysed using RIPA Lysis and Extraction Buffer (89900, ThermoFisher) containing 25 mmol/L Tris-HCl pH 7.6, 150 mmol/L NaCl, 1% NP-40, 1% sodium deoxycholate, 0.1% SDS and Halt™ Protease and Phosphatase Inhibitor Cocktail (78440, ThermoFisher) at 4°C for 20 min. Protein content was quantified using PierceTM BCA Protein Assay Kit (A55864, ThermoFisher). Samples were loaded on 10% Mini-PROTEAN® TGX™ Precast Protein Gels (4561033, Bio-Rad) and then transferred onto PVDF membranes using the Trans-Blot Turbo Mini 0.2 µm PVDF Transfer Pack (1704156, Bio-Rad). Membranes were blocked in 5% skimmed milk for 1 h and incubated with anti-LEMD2 (1:500, HPA017340, Sigma), anti- p44/42 MAPK (Erk1/2) (1:1000, 4695, Cell Signaling), anti-phospho-p44/42 MAPK (Erk1/2) (Thr202/Tyr204) (1:1000, 9101, Cell Signaling), anti-AKT (1:1000, 4691, Cell Signaling), anti-phospho-AKT (Ser473) (1:2000, 4060, Cell Signaling) and anti-Vinculin (1:20000, ab129002, Abcam). Blot quantification was performed using ImageJ (Schneider, Rasband, and Eliceiri 2012).

**Immunocytochemistry**

HepG2 and urothelial cells were seeded and cultured on glass cover slips until 70% confluency. CD19^+^ B-lymphocytes were seeded on glass cover slips directly after isolation by centrifugation of cell suspension at 300xg for 5 min. At endpoint, cells were washed with 1X PBS, fixed with 4% formaldehyde solution (1004969011, Sigma) for 10 min, and permeabilized using 0.1% Triton-X100 (X100, Sigma) in PBS for 5 min. Cells were blocked with 1% BSA in PBS containing 0.1% Triton-X100 for 1 h and subsequently incubated for an additional hour with antibodies against Lamin A/C (1:500, sc-7292, Santa Cruz Biotechnology) and Emerin (1:1000, ab204987, Abcam). Following primary antibody incubation, cells were treated for 1 h (dark) with anti-rabbit IgG Alexa Fluor 549 (1:600, A-11012, ThermoFisher) and anti-mouse IgG Alexa Fluor 488 (1:600, A28175, ThermoFisher) and mounted on microscope slides using Fluoroshield with DAPI (F6057, Sigma). Fluorescent images were acquired using Leica DMi8 inverted microscope (Leica Microsystems). All experimental steps were performed at room temperature.

**Statistical analysis for nuclei counts and Western Blotting**

Data are presented as mean ±SD and statistical analysis was performed with GraphPad Prism Software (San Diego, CA, USA). Details for appropriate statistical testing are presented within the respective figure legend.

**Methods references**

Erden Tayhan, Seçil, Gönül Tezcan Keleş, İsmet Topçu, Erol Mir, and Saime İsmet Deliloğlu Gürhan. 2017. “Isolation and in Vitro Cultivation of Human Urine-Derived Cells: An Alternative Stem Cell Source.” *Turkish Journal of Urology* 43(3):345. doi: 10.5152/TUD.2017.93797.

Schneider, Caroline A., Wayne S. Rasband, and Kevin W. Eliceiri. 2012. “NIH Image to ImageJ: 25 Years of Image Analysis.” *Nature Methods 2012 9:7* 9(7):671–75. doi: 10.1038/nmeth.2089.

**Table S1 Compilation of phenotypic features of the four reported individuals with LEMD2-associated progeroid syndrome**

|  |  | Individual 1  Marbach et al.^1^ | Individual 2  Marbach et al.^1^ | Individual 3  Lu et al.^2^ | **Individual 4**  **(this study)** |
| --- | --- | --- | --- | --- | --- |
| **Development** | Weeks of pregnancy | 36 | 32 | 35 | **37** |
|  | Length at birth | 44 cm | 40.5 cm | 44 cm | **45 cm** |
|  | Head circumference at birth | 31 cm | 26 cm | N/A | **30.5** |
|  | Weight at birth | 1910 g | 1675 g | 1520 g | **2240 g** |
|  | Age of first tooth | 3 y | 7 y (surgically) | 1 y | **4 y** |
|  | White matter hyperintensity | + | N/A | N/A | **+** |
| **Metabolism** | Insulin resistance | + | - | + | **+** |
|  | GH therapy start | 11 y | 4 y | - | **13 y** |
| **Cardiac Profile** | Arterial hypertension | N/A | N/A | N/A | **+** |
|  | Tachycardia | N/A | N/A | N/A | **+** |
| **Facial Features** | Triangular face | +++ | +++ | - | **+++** |
|  | Prominent eyes | +++ | +++ | + | **+++** |
|  | Crooked nose, deviated septum | +++ | + | + | **++** |
|  | Mandibular hypoplasia | +++ | ++ | + | **+++** |
|  | Thin skin, prominent veins | + | ++ | + | **++** |
|  | Generalized lipoatrophy | ++ | + | + | **+++** |
| **Skull & Dentures** | Microcephaly | +++ | ++ | - | **+++** |
|  | Wormian bones | +++ | +++ | + | **+++** |
|  | Open cranial sutures | - | - | N/A | **+** |
|  | Dental crowding | +++ | +++ | - | **-** |
|  | Supernumerary teeth | +++ | +++ | N/A | **-** |
|  | Delayed dentition | +++ | +++ | + | **+++** |
| **Skeletal Features** | Short stature | +++ | - | - | **+++** |
|  | Low bone density | ++ | - | N/A | **++** |
|  | Hypoplastic clavicles | ++ | ++ | - | **-** |
|  | Exostoses | N/A | N/A | N/A | **+++** |
|  | Intention tremor | ++ | ++ | - | **-** |

N/A: Information not available

^1^ Marbach, F., Rustad, C. F., Riess, A., Đukić, D., Hsieh, T. C., Jobani, I., Prescott, T., Bevot, A., Erger, F., Houge, G., Redfors, M., Altmueller, J., Stokowy, T., Gilissen, C., Kubisch, C., Scarano, E., Mazzanti, L., Fiskerstrand, T., Krawitz, P. M., Lessel, D., Netzer, C. (2019). The Discovery of a LEMD2-Associated Nuclear Envelopathy with Early Progeroid Appearance Suggests Advanced Applications for AI-Driven Facial Phenotyping. *American journal of human genetics*, 104(4), 749–757. https://doi.org/10.1016/j.ajhg.2019.02.021

^2^ Lu, Z., Zhang, W., Mao, X., Li, D., Chen, X., Liu, L., & Lin, Y. (2023). The third case of Marbach-Rustad progeroid syndrome caused by a de novo LEMD2 variant. *Clinical genetics*, 10.1111/cge.14441. Advance online publication. https://doi.org/10.1111/cge.14441

**Table S2 Compilation of measured clinical markers in our patient with LEMD2-associated progeroid syndrome**

| **Patient age (y)** | **VitD-P (nmol/L)** | **ALT-P (U/L)** | **HDL-P (mmol/L)** | **TG-P (mmol/L)** | **GGT-P (U/L)** | **HAST-P (mmol/L)** | **ALP-P (U/L)** | **AST-P (U/L)** | **GLU-P (mmol/L)** | **HOMA-IR** | **LDH-P (U/L)** | **Insulin (pmol/L)** | **Lactate (mmol/L)** | **Bilirubin (μmol/L)** | **Uric acid (μmol/L)** | **Biotinidase (nmol/min/mL)** | **LDL (mmol/L)** | **Albumin (g/L)** | **Creatinine (μmol/L)** | **Weight (kg)** | **BMI** |
| --- | --- | --- | --- | --- | --- | --- | --- | --- | --- | --- | --- | --- | --- | --- | --- | --- | --- | --- | --- | --- | --- |
| 13 |  | 22 |  | 1 |  |  | 132 | 25 | 4.7 | 7.5 |  | 36.1 |  |  | 397 |  |  |  |  |  |  |
| 13 |  | 20 |  | 2.2 |  |  | 90 | 20 |  |  |  |  |  |  | 403 |  |  |  |  | 26.1 | 15.37 |
| 14 | 17.5 | 66 |  |  | 50 | 3.9 |  | 40 | 5.3 |  | 315 | 568 |  | 4 |  | 14.2 |  |  |  | 27 |  |
| 15 | 84.8 | 32 | 0.77 | 0.79 | 21 |  | 69 | 27 | 4.6 |  | 161 |  | 2 |  | 252 | 10.5 | 1.75 |  |  | 22.7 | 13.3 |
| 15 | 105 | 27 |  | 0.66 |  |  |  | 16 | 4.6 | 12.6 |  | 427 | 1.4 | 7 | 301 |  |  |  |  | 26.6 | 15.45 |
| 16 | 80 | 30 |  | 0.88 | 37 |  |  | 17 | 4.6 | 17.6 |  | 596 | 1.9 |  | 373 |  |  |  |  | 27.1 | 15.67 |
| 17 | 59.1 | 46 |  | 1.9 |  |  |  | 26 | 4.3 | 6.8 |  |  | 4 |  | 246 |  |  | 46 | 27 | 27.8 | 16.07 |
| 17 |  | 260 |  |  | 287 |  |  | 306 | 7.8 |  |  |  |  | 11 |  |  |  |  |  | 27 | 15.73 |
| 17 |  | 135 |  |  | 192 | 2.2 |  | 43 |  |  |  | 55 |  |  |  |  |  |  | 31 |  |  |
| 17 | 77.9 | 41 |  | 1.7 |  |  |  | 17 | 5.2 | 1.6 |  | 528 | 3.2 |  |  | 8.6 |  |  |  | 27 | 15.61 |
| 17 | 54.7 | 25 |  | 0.7 | 18 |  |  | 14 | 4.2 |  |  |  | 4.8 |  |  |  |  |  |  |  |  |
| 17 |  | 33 |  | 4.3 |  |  |  | 19 | 4.3 |  |  |  |  |  |  |  |  |  |  |  |  |
| 18 | 88.6 | 68 |  | 3.8 | 58 | 2.7 |  | 43 | 4.8 |  |  | 265 | 4.1 |  |  |  |  |  |  | 27.1 | 15.82 |
| 18 |  | 53 |  | 3.4 | 70 |  |  | 28 | 4.6 |  |  | 222 | 3.5 |  |  | 18.7 |  |  |  | 27.8 | 16.07 |
| 18 | 108.3 | 73 |  | 5.6 | 67 |  |  | 51 | 4.5 |  |  | 382 | 5.2 |  |  |  |  |  |  | 27.2 | 15.82 |
| 18 |  | 44 | 0.6 | 6.9 | 56 |  |  | 36 | 4.9 |  |  | 277 | 2.9 |  |  |  |  |  |  | 26.2 | 15.22 |
| 19 | 82.2 | 41 |  | 3.3 | 47 | 3.4 |  | 20 | 5.1 |  | 120 | 271 | 3.3 |  |  |  |  |  |  | 23.6 | 13.68 |
| 19 |  | 56 |  | 7.7 | 64 |  | 47 | 37 | 4.9 |  |  | 208 | 2.8 |  |  | 18.5 |  |  |  | 26.2 | 15.08 |
| 19 |  | 60 | 0.5 | 9.1 | 58 |  | 42 | 37 | 4.3 |  |  | 253 |  |  |  |  |  |  |  | 26.4 | 15.29 |
| 19 | 80.8 | 59 |  | 5 | 58 |  |  | 46 | 4.4 |  |  | 193 | 3.7 |  |  |  |  |  |  | 26.1 | 15.16 |
| 19 | 86.4 | 97 | 0.6 | 5.4 | 92 |  |  | 91 | 4.9 |  |  | 295 | 4.3 |  |  |  |  |  |  | 26.8 | 15.54 |
| 19 |  | 95 |  | 3.5 |  | 2.5 |  | 53 | 5.4 |  | 169 | 264 | 3.6 |  |  |  |  |  |  |  |  |
| 20 | 86.8 | 61 |  | 4 | 82 |  |  | 42 | 4.3 |  |  |  | 3.3 |  |  |  |  |  |  | 26.9 | 15.55 |
| 20 |  | 74 |  | 6 | 61 | 2.9 | 42 | 61 | 4.6 |  |  |  | 3.7 |  |  |  |  |  |  | 24.3 | 14.03 |
| 20 | 78.1 | 47 |  | 5.3 | 98 |  | 53 | 42 | 3.9 |  |  | 551 | 2.8 |  |  | 16.9 |  |  |  | 26.3 | 15.23 |
| 20 | 90.2 | 61 |  | 4.7 | 84 |  |  | 66 | 4.2 |  |  | 230 | 2.4 |  |  | 16.4 |  |  |  | 25.7 | 14.9 |
| 21 |  | 60 |  | 5.6 | 117 |  |  | 41 | 4.9 |  |  | 557 | 2.6 |  |  | 14 |  |  |  | 26.2 | 15.15 |
| 21 |  | 49 |  | 7.8 | 99 | 2.5 |  | 40 | 4.8 |  |  | 749 | 2.7 |  |  |  |  |  |  | 26.03 | 15.23 |
| 21 | 42 | 52 |  | 5.6 | 93 |  |  | 33 | 4.6 |  | 130 | 472.7 | 2.6 |  |  | 15.2 |  |  |  | 25.7 | 14.93 |
| 22 | 89.2 | 89 |  | 5.4 | 114 |  |  | 84 | 5.8 | 31.7 |  | 868.5 | 3.2 |  |  |  |  |  |  | 27 | 15.61 |
| 22 | 69.4 | 78 | 0.6 | 2.3 | 77 |  |  | 61 | 4.7 |  |  | 204.8 | 3.4 |  |  | 15.8 | 2.8 |  | 24 |  |  |
| 23 | 74.7 | 56 | 0.7 | 3.1 | 65 |  |  | 34 | 4.2 | 29.9 |  | 1087 | 2.6 |  |  | 15.7 | 2.3 |  |  | 25 | 14.45 |
| 23 | 71.4 | 88 |  | 3.3 |  | 2.2 | 44 | 56 | 4.8 | 21.7 | 154 | 706.9 | 3.5 |  |  | 13.8 |  |  |  | 26.4 | 15.26 |
| 24 | 45 | 111 | 0.6 | 4.4 | 77 | 3.2 | 38 | 103 | 3.8 |  |  | 303.7 | 3.6 |  |  | 12.9 | 2.1 |  |  |  |  |
| 25 | 304.4 | 47 | 0.7 | 3.7 | 55 | 3.4 | 48 | 33 | 3.7 | 4.1 |  | 172.2 | 3.1 |  |  | 12.9 |  |  |  | 23.9 | 13.86 |

Abbreviations: ALP, Alkaline phosphatase; ALT, Alanine transaminase; AST, Aspartate transaminase; GGT, Gamma-glutamyltransferase; HAST-P, Spot urea; HDL-P, High-density lipoprotein particle concentration; HOMA-IR, Homeostatic Model Assessment for Insulin Resistance; LDH-P, Lactate dehydrogenase; LDL, Low-density lipoprotein; TG-P, Triglycerides; VitD-P, Vitamin D.

**Figure S1. 3D skull reconstruction of our patient with LEMD2-associated progeroid syndrome.** CT scan confirming the presence of multiple bilateral switch bones in the lambdoid suture (Wormian bones, yellow arrows).


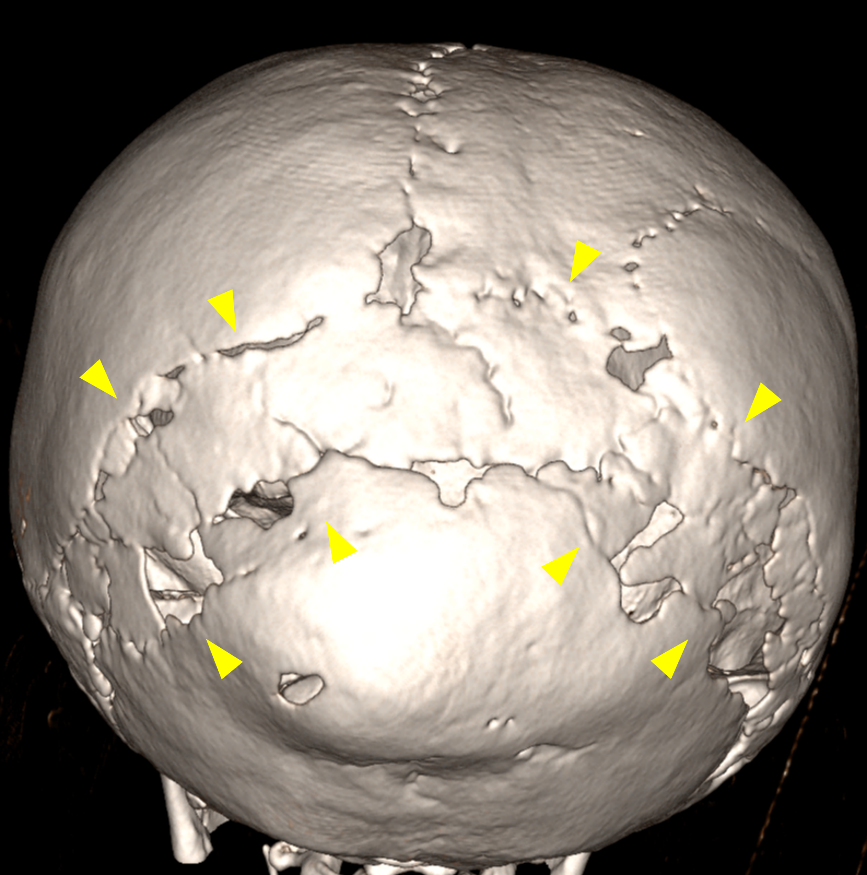


**Figure S2. Time course of relevant biochemical markers obtained from our patient with LEMD2-associated progeroid syndrome.** Patient ALT and AST liver enzymes, indicative of liver cell integrity (A), insulin levels and corresponding metformin treatment (B), and biotinidase activity and triglyceride concentration (C) were measured during the last 10 years (ages 13-24) during clinical visits. In (A and C), the dotted lines correspond to the upper normal ALT (< 41 U/L) and biotinidase (10.6 nmol/min/mL) reference levels, while in (A, B and C), the dashed lines correspond to upper normal AST (< 27 U/L), insulin (173 pmol/L), and triglyceride (2 mmol/L) reference levels, respectively.


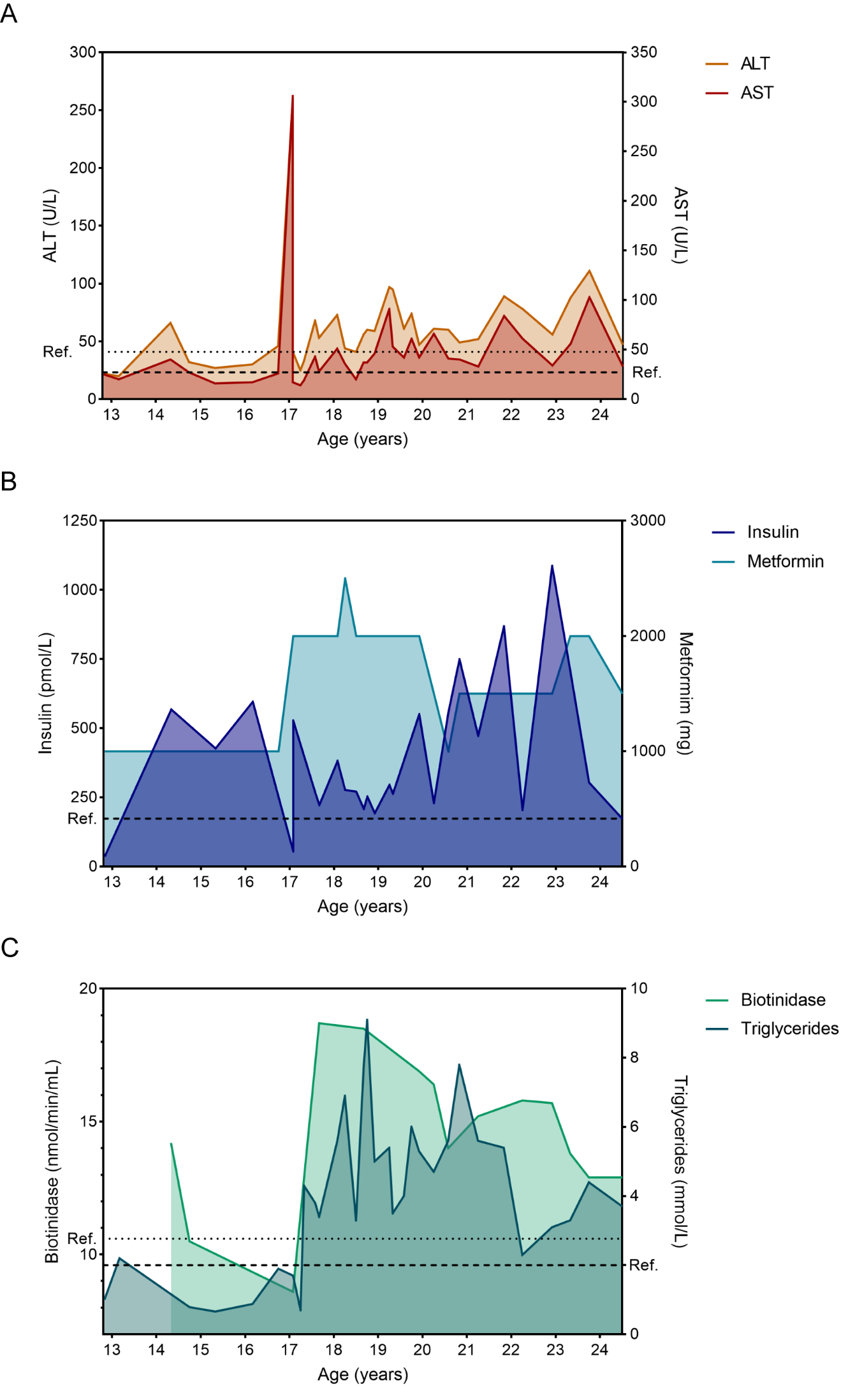


**Figure S3. Nuclear circularity.** Quantification of nuclear circularity from HUCs (WT, n=130; LEMD2^mut^, n=115), CD19^+^ B cells (WT, n=242; LEMD2^mut^, n=217) and HepG2 cells (siCtrl, n=219; siLEMD2, n=236) was performed using Shape Descriptor function of ImageJ (Schneider, Rasband, and Eliceiri 2012) that defines circularity as 4π*area/perimeter^2. A value of 1.0 indicates a perfect circle, whereas values approaching 0.0 indicate shape elongation. Mean circularity value of counted nuclei ± SD of three independent experiments (t-test, ∗ = p ≤ 0.05, ∗∗ = p ≤ 0.01).


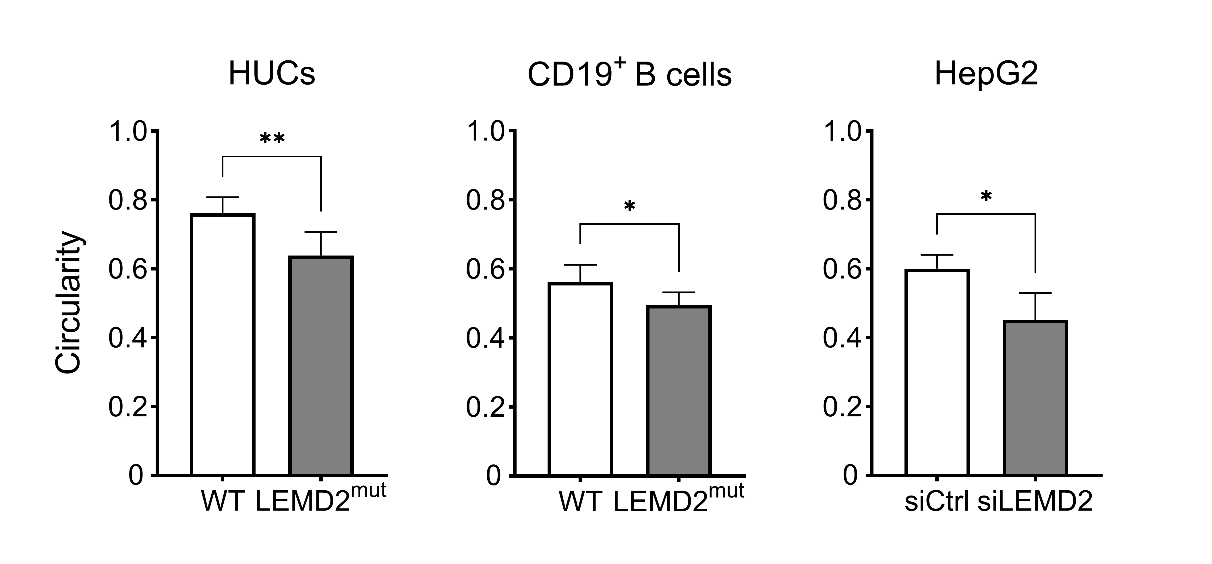


**Figure S4. Representative Western Blot and band intensity quantification graph for monitoring expression of AKT, ERK1/2 and pAKT and pERK1/2 in HepG2 cells at 96 h post-transfection with either siCtrl or siLEMD2.** Quantification performed for the phosphorylated proteins. Mean ± SD of three independent experiments (t-test, ns = non-significant, ∗ = p ≤ 0.05).


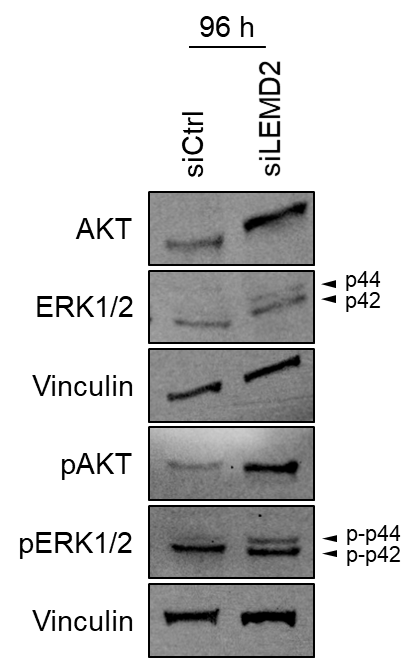

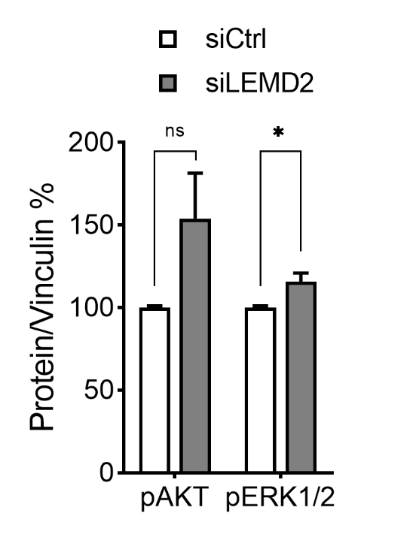

Supplement: Supplementary file 1 — Figure S1. Figure S2. Figure S3. Figure S4. Table S1. Table S2. [file ACEL-23-e14189-s001.zip › acel14189-sup-0001-Supplementary Material.docx]
