# Supplementary figures and images for: LEMD2‐associated progeroid syndrome: Expanding the phenotype of the nuclear envelopathy caused by a defect in LEMD2 gene
Source: Aging Cell. 2024 May 16;23(8):e14189. doi: 10.1111/acel.14189 (PMC11320348; doi:10.1111/acel.14189)

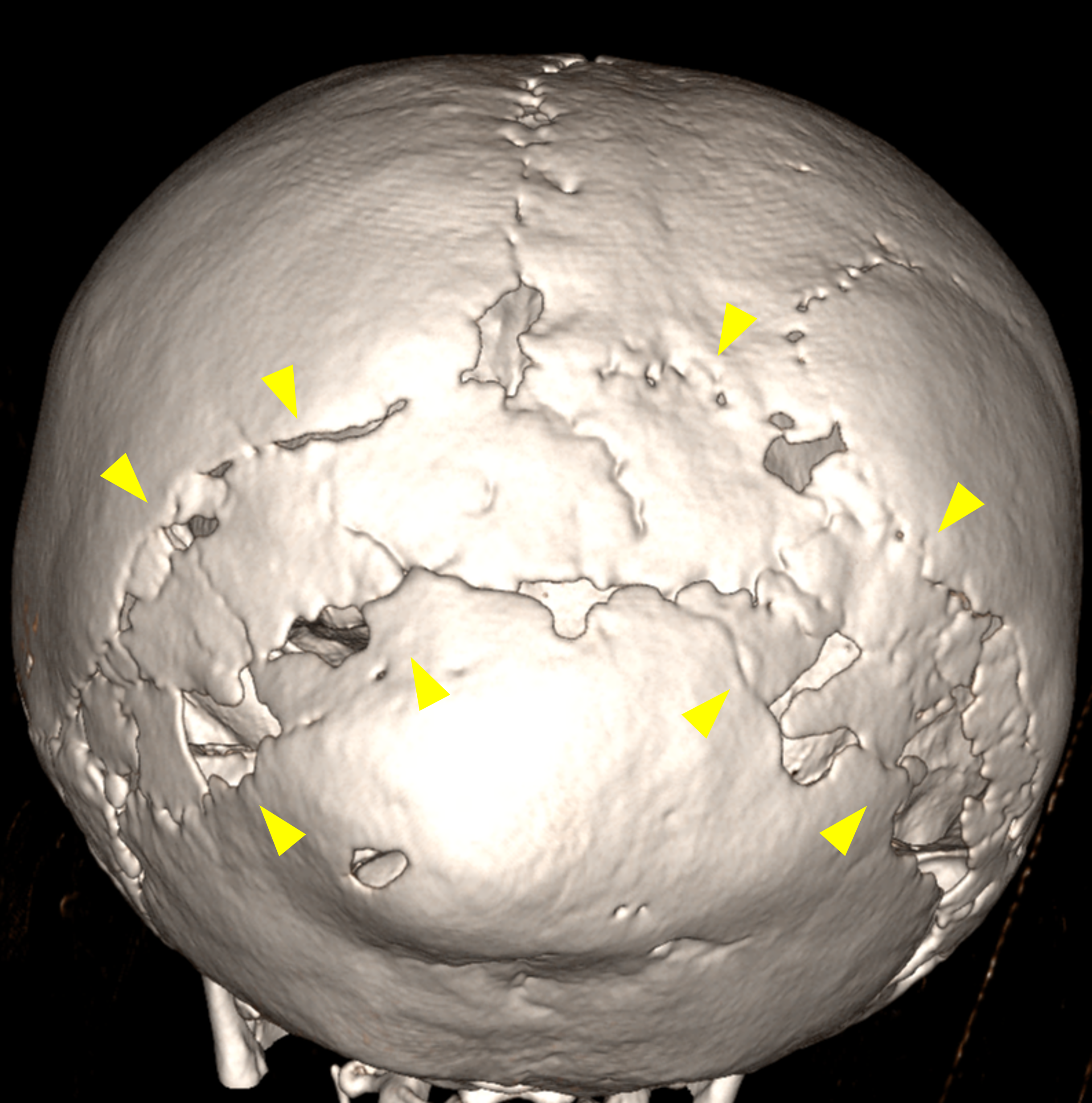

Supplement: Supplementary file 1 — Figure S1. Figure S2. Figure S3. Figure S4. Table S1. Table S2. [file ACEL-23-e14189-s001.zip › ACEL_14189-sup-1_Figure S1.PNG]

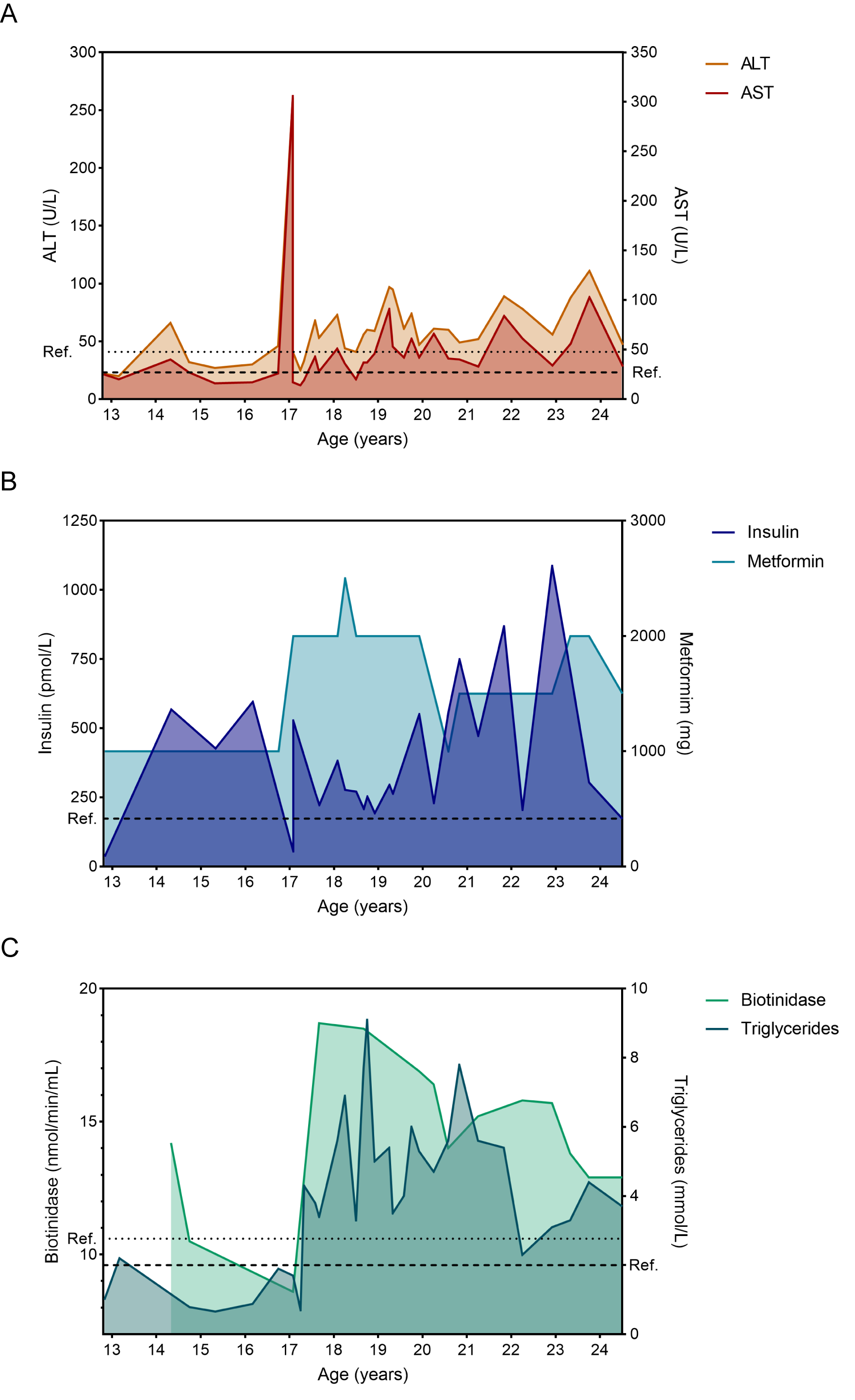

Supplement: Supplementary file 1 — Figure S1. Figure S2. Figure S3. Figure S4. Table S1. Table S2. [file ACEL-23-e14189-s001.zip › ACEL_14189-sup-2_Figure S2.tif]

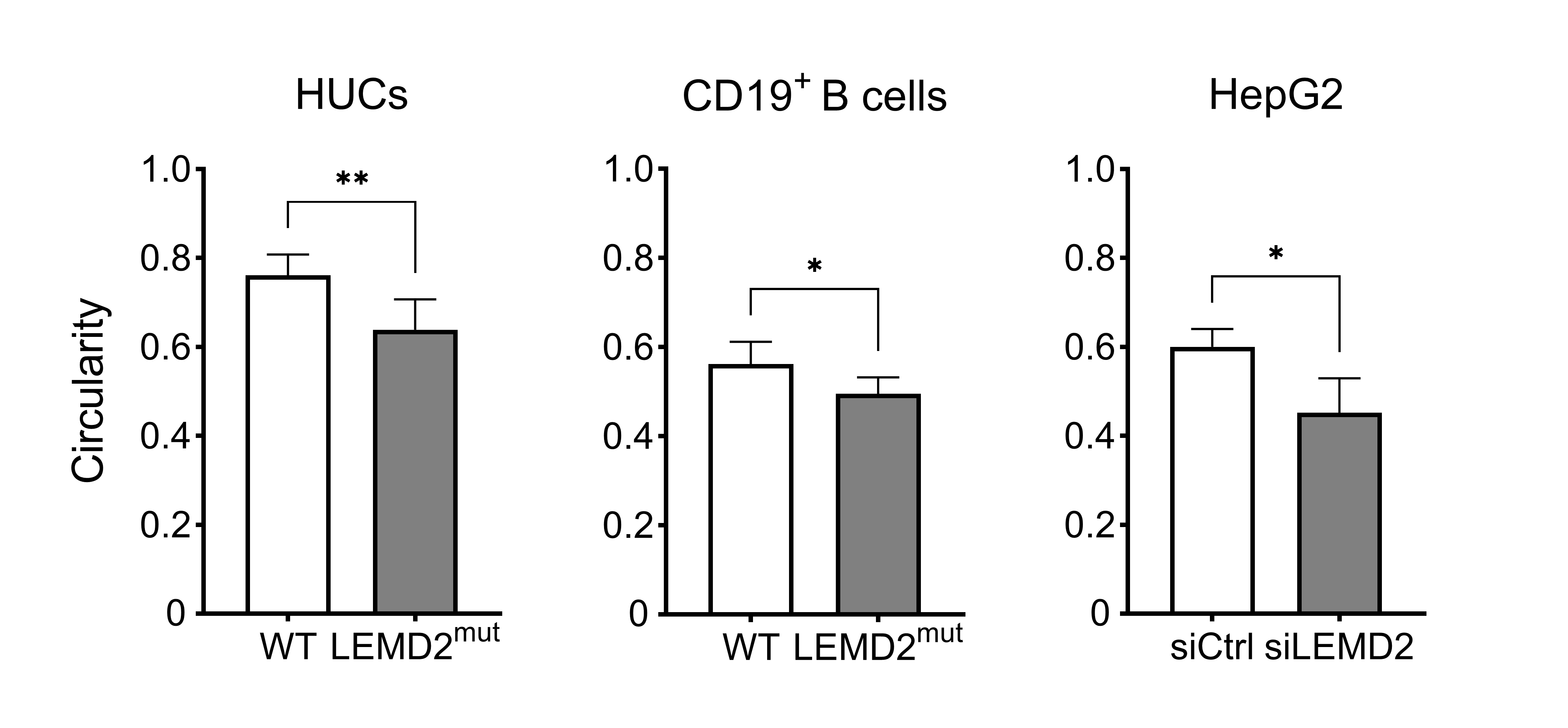

Supplement: Supplementary file 1 — Figure S1. Figure S2. Figure S3. Figure S4. Table S1. Table S2. [file ACEL-23-e14189-s001.zip › ACEL_14189-sup-3_Figure S3.tif]

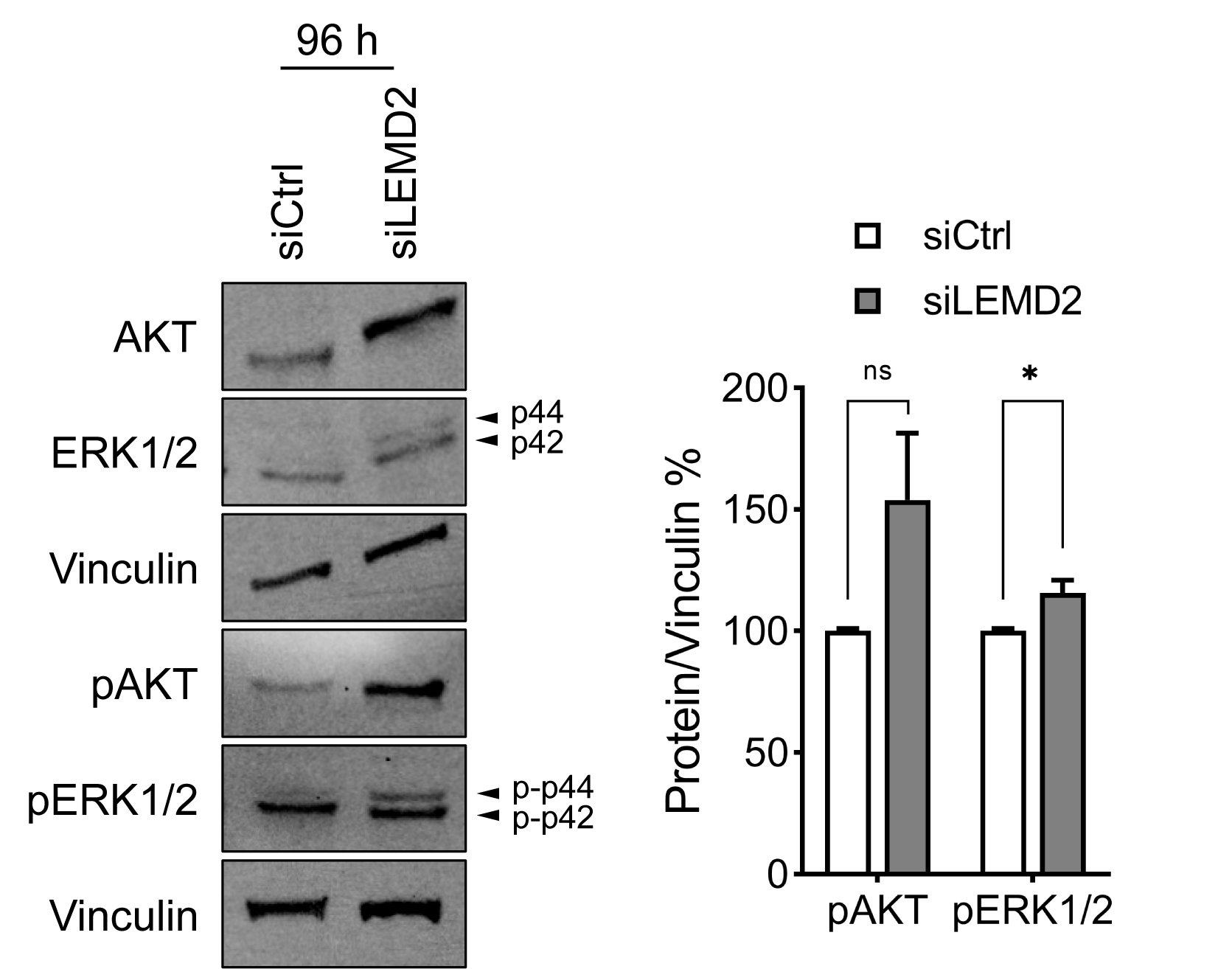

Supplement: Supplementary file 1 — Figure S1. Figure S2. Figure S3. Figure S4. Table S1. Table S2. [file ACEL-23-e14189-s001.zip › ACEL_14189-sup-4_Figure S4.tif]
